# PD-L1 deficiency exacerbates colitis severity by remodeling gut microbiota in inflammatory bowel disease

**DOI:** 10.3389/fimmu.2025.1622744

**Published:** 2025-06-30

**Authors:** Yixian Ma, Jinshan Suo, Siqi Sheng, Ling Chen

**Affiliations:** ^1^ Eye Institute and Department of Ophthalmology, Eye & ENT Hospital, Fudan University, Shanghai, China; ^2^ Shanghai Key Laboratory of Visual Impairment and Restoration, Shanghai, China; ^3^ NHC Key Laboratory of Myopia and Related Eye Diseases, Key Laboratory of Myopia and Related Eye Diseases, Chinese Academy of Medical Sciences, Shanghai, China; ^4^ Department of Immunology, School of Cell and Gene Therapy, Songjiang Research Institute, Shanghai Songjiang District Central Hospital, Shanghai Jiao Tong University School of Medicine, Shanghai, China; ^5^ Ophthalmology Medical Center, The First Affiliated Hospital of Chongqing Medical University, Chongqing Key Laboratory for the Prevention and Treatment of Major Blinding Eye Diseases, Chongqing Branch (Municipality Division) of National Clinical Research Centre for Ocular Diseases, Chongqing, China

**Keywords:** programmed death ligand 1 (PD-L1), gut microbiota, metagenomic analysis, autoimmunity, inflammatory bowel disease (IBD)

## Abstract

**Background:**

Inflammatory bowel disease (IBD) is a chronic autoimmune disorder driven by gut microbiota dysbiosis. As an essential immune checkpoint, Programmed death-ligand 1 (PD-L1) has been implicated in modulating gut microbiota composition. However, the precise role of PD-L1 in shaping metagenomic profiles during IBD-associated colitis remains unexplored.

**Methods:**

DSS-induced colitis was established in both PD-L1 knockout (*Pdcd1lg1-/-*) mice and wild-type (wt) control mice. Clinical parameters, including disease activity index (DAI), body weight changes, colon length, and histopathological alterations, were systematically evaluated using non-parametric Kruskal-Wallis tests and ANOVA to compare colitis severity between genotypes.

**Results:**

PD-L1 knockout mice exhibited exacerbated colitis, manifesting significantly greater weight loss (p<0.05 vs. wt_DSS), colonic shortening (p<0.05), and DAI scores (p<0.05) and inflammatory changes. PD-L1 knockout mice showed distinct dysbiosis, with enriched pathobionts (*Escherichia coli*, p=0.006; *Bacteroides thetaiotaomicron*, p=0.015) and depletion of commensals (*Tritrichomonas foetus*, p<0.001; *Ligilactobacillus murinus*). Alpha diversity analysis using Chao1 index revealed statistically significant differences between experimental groups (p=0.05). The transporters downregulate anti-inflammatory SCFA metabolism. KEGG enrichment analysis of differentially expressed genes (DEGs) revealed significant associations with immune and inflammatory pathways in PD-L1 knockout mice.

**Conclusion:**

PD-L1 deficiency aggravates colitis by driving pathogenic microbiota alterations and impairing microbial metabolic homeostasis, highlighting its dual regulatory roles in immune homeostasis and microbiome dynamics.

## 1 Introduction

Inflammatory bowel disease (IBD), encompassing Crohn’s disease (CD) and ulcerative colitis (UC), is a chronic and relapsing inflammatory disorder of the gastrointestinal tract with rising global incidence, particularly in industrialized nations (1, 2). IBD is more common in young and middle-aged adults, but the incidence has been increasing in children and the elderly in recent years. Some studies show that the male-to-female ratio for UC is nearly equal, while CD is slightly more prevalent in females (2, 3).

Environmental triggers such as dietary patterns, pharmacological exposures, and lifestyle factors significantly impact gut microbiota homeostasis. Excessive consumption of high-fat/low-fiber diets and inappropriate antibiotic use have been shown to disrupt microbial symbiosis, resulting in diminished production of beneficial metabolites including short-chain fatty acids (SCFAs) while simultaneously impairing intestinal epithelial barrier integrity (4, 5). Epidemiological studies highlight that the prevalence of IBD exhibits notable geographical variations. For example, gut microbiota profiles of patients from Mexico and Spain differ significantly due to dietary differences, and microbiome characteristics in Middle Eastern populations may influence IBD risk through specific dietary patterns, such as high-fiber and fermented foods consumption (6, 7).

The pathogenesis of IBD involves complex multifactorial interactions, primarily including genetic susceptibility, mutations in specific genes (e.g., NOD2, IL23R) increase the risk of intestinal barrier defects or aberrant immune responses (1, 2). Immune dysregulation in IBD involves aberrant immune responses against gut microbiota, characterized by excessive production of pro-inflammatory cytokines such as TNF-α, IL-6, and IL-17, coupled with insufficient anti-inflammatory mediators including IL-10 (8–10). Intestinal barrier disruption primarily arises from impaired epithelial tight junction complexes, particularly involving occludin and claudin proteins, which compromise mucosal integrity and facilitate microbial translocation, thereby triggering innate immune activation through the leaky gut mechanism (10, 11).

Notably, the gut microbiota has emerged as a pivotal contributor, where dysbiosis—characterized by reduced microbial diversity, depletion of commensal bacteria (e.g., *Faecalibacterium prausnitzii*), and expansion of pathobionts (e.g., *Escherichia coli*)—disrupts mucosal homeostasis and drives aberrant immune activation (12, 13). Altered microbial metabolites (e.g., secondary bile acids, hydrogen sulfide) disrupt redox homeostasis and potentiate pro-inflammatory responses via oxidative stress-mediated pathways (7, 14).

The programmed death-ligand 1 (PD-L1), encoded by the gene *Pdcd1lg1*, is a critical immune checkpoint protein that interacts with programmed death-1 (PD-1) on T cells. This interaction inhibits T cell activation and proliferation, thereby preventing excessive immune responses. It also promotes the differentiation of regulatory T cells (Tregs) and induces effector T-cell exhaustion, maintaining immune tolerance. Additionally, it modulates inflammatory cytokines such as IFN-γ and IL-2 (15, 16). In the intestinal microenvironment, PD-L1 exhibits context-dependent functions. The gut microbiota composition directly modulates PD-1/PD-L1 inhibitor efficacy, where specific probiotics, such as *Bifidobacterium*, enhance anti-PD-1 therapeutic response through PD-L2 downregulation (17). Studies demonstrate that intestinal epithelial cells upregulate PD-L1 following stimulation with gut microbial metabolites (e.g., L-DNA), promoting activated Th2 cell apoptosis to modulate local intestinal immune responses (18). PD-L1 deficiency disrupts gut microbiota homeostasis and exacerbates DSS-induced colitis (19). In contrast, PD-L1 attenuates intestinal graft injury through innate immune response suppression during intestinal transplantation, demonstrating its essential role in preserving intestinal immune homeostasis (20, 21).

Although studies on gut microbiota in IBD and the association between PD-L1 and microbiota are increasing, systematic investigations into the gut microbiota in PD-L1 deficiency-mediated colitis remain insufficient. This study aims to explore the impact of *Pdcd1lg1* knockout on gut microbiota composition in C57BL/6J mice under inflammatory bowel disease conditions. It systematically investigates the effects of PD-L1 genetic ablation on gut microbiota composition in C57BL/6J mice under experimental colitis conditions.

## 2 Materials and methods

### 2.1 Animals and experimental design

Female C57BL/6J mice and female *Pdcd1lg1* knockout C57BL/6J mice (*Pdcd1lg1 -/-* mice), aged 6–8 weeks, were obtained from Cyagen Biosciences, Suzhou, China. All experimental procedures were performed according to the guidelines of the Animal Care and Use Committee of Fudan University (Shanghai, China). Animals were housed at the Experimental Animal Center of the Eye and ENT Hospital of Fudan University with a temperature of 22-26°C, 12-hour light/dark cycles, and relative humidity of 40-70%.

**Figure 1:**
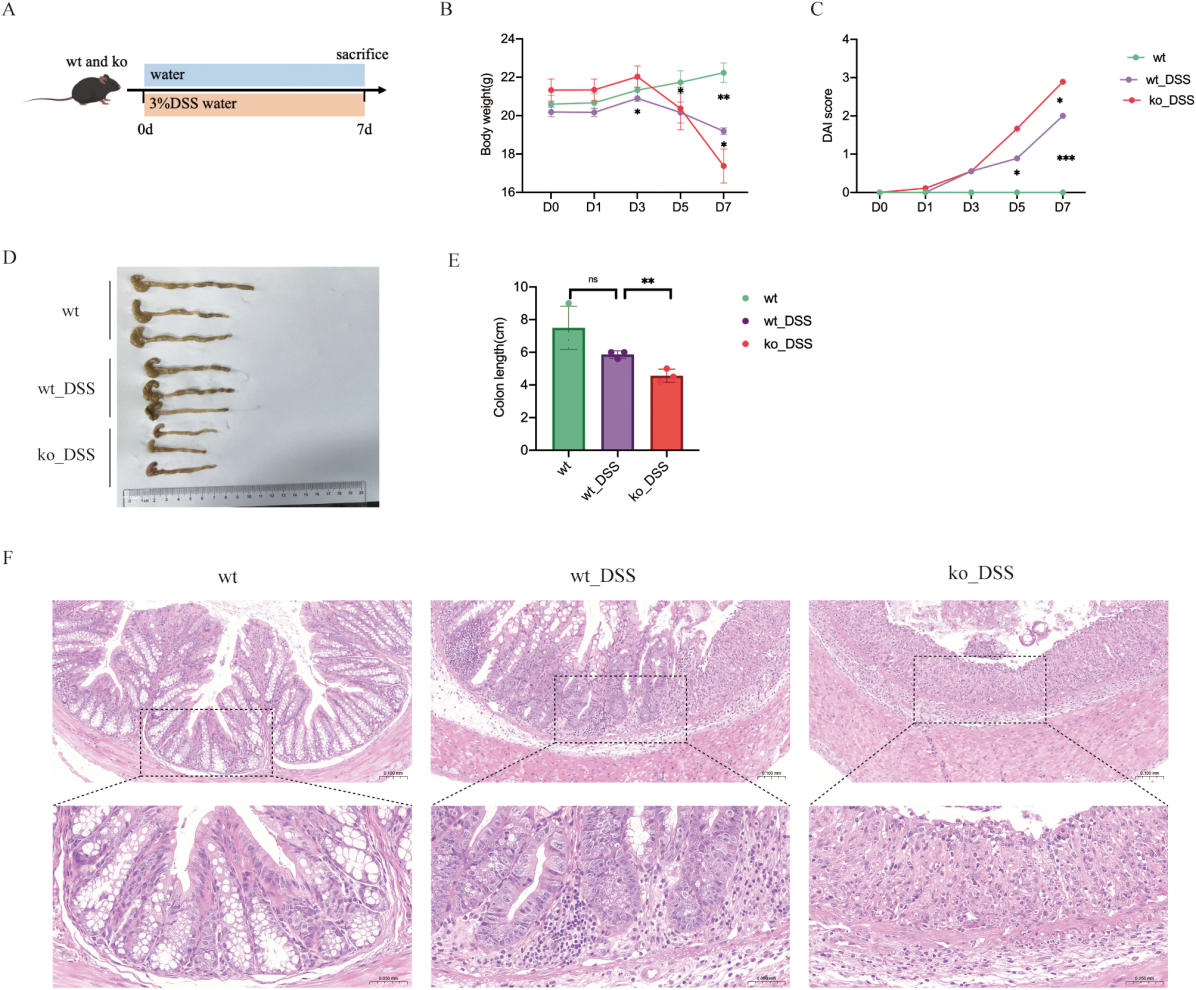
**(A)** Experimental protocol for DSS-induced colitis mice model. **(B)** Body weight change of mice during modeling period. **(C)** DAI change. **(D)** Photographs of representative colons of each group. **(E)** Average length of colons of each group. **(F)** Histopathological analysis of colonic tissues. *p < 0.05; **p < 0.01; ***p < 0.001; ns, no significance.

Then, animals were randomly divided into four groups (n=3/group) including wt_control (wild-type without DSS), ko_control (*Pdcd1lg1* -/- without DSS), wt_DSS (wild-type with DSS-induced colitis), and ko_DSS (*Pdcd1lg1* -/- with DSS-induced colitis) (Dextran Sulfate Sodium, Yeasen biotech Co., Ltd., Shanghai, China). Colitis was induced in mice by daily administration of 3% DSS for 7 days, while the control group received equivalent volumes of sterile distilled water (**Figure 1A**). Body weights were recorded at baseline (day 0) and on day1, 3, 5, and 7 post-modeling. On day 7, mice were sacrificed via cervical dislocation under anesthesia (1.25% bromethol, 0.2 mL/10g body weight administered intraperitoneally; Aibei Biotechnology Co., Ltd., Nanjing, China). Collect fecal specimens and obtain colonic samples extending from the cecum junction to the anal verge. Immediately after collection, the fecal specimens were flash-frozen in liquid nitrogen and then stored at -80°C to maintain microbial viability and DNA integrity for subsequent sequencing analysis. Colon length was measured along the mesenteric border before fixation in 4% paraformaldehyde, followed by paraffin embedding and hematoxylin & eosin (H&E) staining. The disease activity index (DAI) was calculated to assess the severity of UC in animal models in light of the study (22). DAI = (Weight loss score + Stool score + Bleeding score)/3.

### 2.2 Metagenome DNA extraction and shotgun sequencing

Total microbial genomic DNA was extracted from fecal samples using the OMEGA Mag-Bind Soil DNA Kit (M5635-02; Omega Bio-Tek, GA, USA) according to the manufacturer’s protocol, and stored at -20°C until further assessment. DNA concentration and purity were quantified using a Qubit™ 4 Fluorometer (Thermo Fisher Scientific Inc., MA, USA), while integrity was verified by agarose gel electrophoresis. The extracted microbial DNA was processed to construct metagenome shotgun sequencing libraries with insert sizes of ~400 bp by using Illumina TruSeq Nano DNA LT Library Preparation Kit (Illumina, CA, USA). Each library was sequenced by Illumina NovaSeq platform (Illumina, CA, USA) with PE150 strategy at Personal Biotechnology Co., Ltd. (Shanghai, China).

### 2.3 Metagenomics analysis

Raw sequencing reads were processed through adapter removal with Cutadapt (v1.2.1) (23), sliding-window quality trimming using fastp (v0.23.2) (24), and host DNA removal by alignment to the mouse genome via Minimap2 (v2.24-r1122) to generate quality-filtered data (25). Once quality-filtered reads were obtained, taxonomical classifications of metagenomics sequencing reads from each sample were performed using Kraken2 (v2.0.8 beta) (26) against databases including NCBI-nt, GTDB and RVDB. Reads assigned to metazoans or viridiplantae were removed for downstream analysis. Megahit (v1.1.2) (27) was used for assembly of reads in each sample using the meta-large presetted parameters. The generated contigs (longer than 300bp) were then pooled together and clustered using MMseqs2 (version 13.45111) (28) with “easy-linclust” mode, setting sequence identity threshold to 0.95 and covered residues of the shorter contig to 90%. The lowest common ancestor taxonomy of the non-redundant contigs was obtained by aligning them against the NCBI-nt database by MMseqs2 with “taxonomy” mode, and contigs assigned to Viridiplantae or Metazoa were dropped in the following analysis. Prodigal (V2.6.3) (29) was used to predict the genes in the contigs. CDS sequences of all samples were clustered by MMseqs2 with “easy-cluster” mode, setting protein sequence identity threshold to 0.95 and covered residues of the shorter contig to 90%. To assess the abundances of these genes, the high-quality reads from each sample were mapped onto the predicted gene sequences using Minimap2 with “-ax sr –sam-hit-only” and using featureCounts to count the number of reads aligned to gene sequences, i.e., the Reads Count (RC) (30) for each gene. The functionality of the non-redundant genes was obtained by annotated using MMseqs2 with the “search” mode against the protein databases of KEGG.

### 2.4 Statistical analyses

In this study, statistical tests (Kruskal-Wallis test, ANOVA) were conducted using R software (version 3.6.1). Based on the taxonomic and functional profiles of non-redundant genes, LEfSe (Linear discriminant analysis effect size) was performed to detect differentially abundant taxa and functions across groups using the default parameters (31). Beta diversity analysis was performed to investigate the compositional and functional variation of microbial communities across samples using Bray-Curtis distance metrics (32) and visualized via principal coordinate analysis (PCoA), nonmetric multidimensional scaling (NMDS) and unweighted pair-group method with arithmetic means (UPGMA) hierarchical clustering (33). P < 0.05, P < 0.01 and P < 0.001 were considered significant. GraphPad Prism 9.0 software (GraphPad Software Inc, La Jolla, CA, USA) was used to draw images.

## 3 Results

### 3.1 PD-L1 knockout mice had more severe intestinal inflammation

All mice exhibited normal activity, dietary intake, stool consistency, and weight gain before modelling. In comparison with those in the control group, the mice in the model group showed not only significantly decreased body weight (**Figure 1B**) and colon length (**Figures 1D, E**), but also observably increased DAI scores (**Figure 1C**). Both wt_DSS and ko_DSS groups experienced significant weight loss compared to their respective controls (p<0.05). Notably, the ko_DSS group showed more pronounced weight loss than the wt_DSS group (p<0.05), indicating exacerbated colitis in *Pdcd1lg1 -/-* mice (**Figure 1B**). The wt_DSS group had shorter colons than the wt control (p<0.05), reflecting colitis-induced shortening. The ko_DSS group exhibited the shortest colons (p<0.05 vs. wt_DSS), suggesting more severe colitis in *Pdcd1lg1 -/-* mice (**Figures 1D, E** **).** Both wt_DSS and ko_DSS groups had higher DAI scores than wt group (p<0.05). The ko_DSS group had the highest DAI scores (p<0.05 vs. wt_DSS), indicating the most severe colitis. In addition, the macroscopic morphology of colonic tissues showed colon edema, a reduced caecal volume and an enteric cavity with bloody stools. The ko_DSS group mice exhibited severed colonic shortening and colonic edema than both wt group mice and wt_DSS group mice (**Figure 1F**).

### 3.2 The gut microbiota in four groups is altered

**Figure 2:**
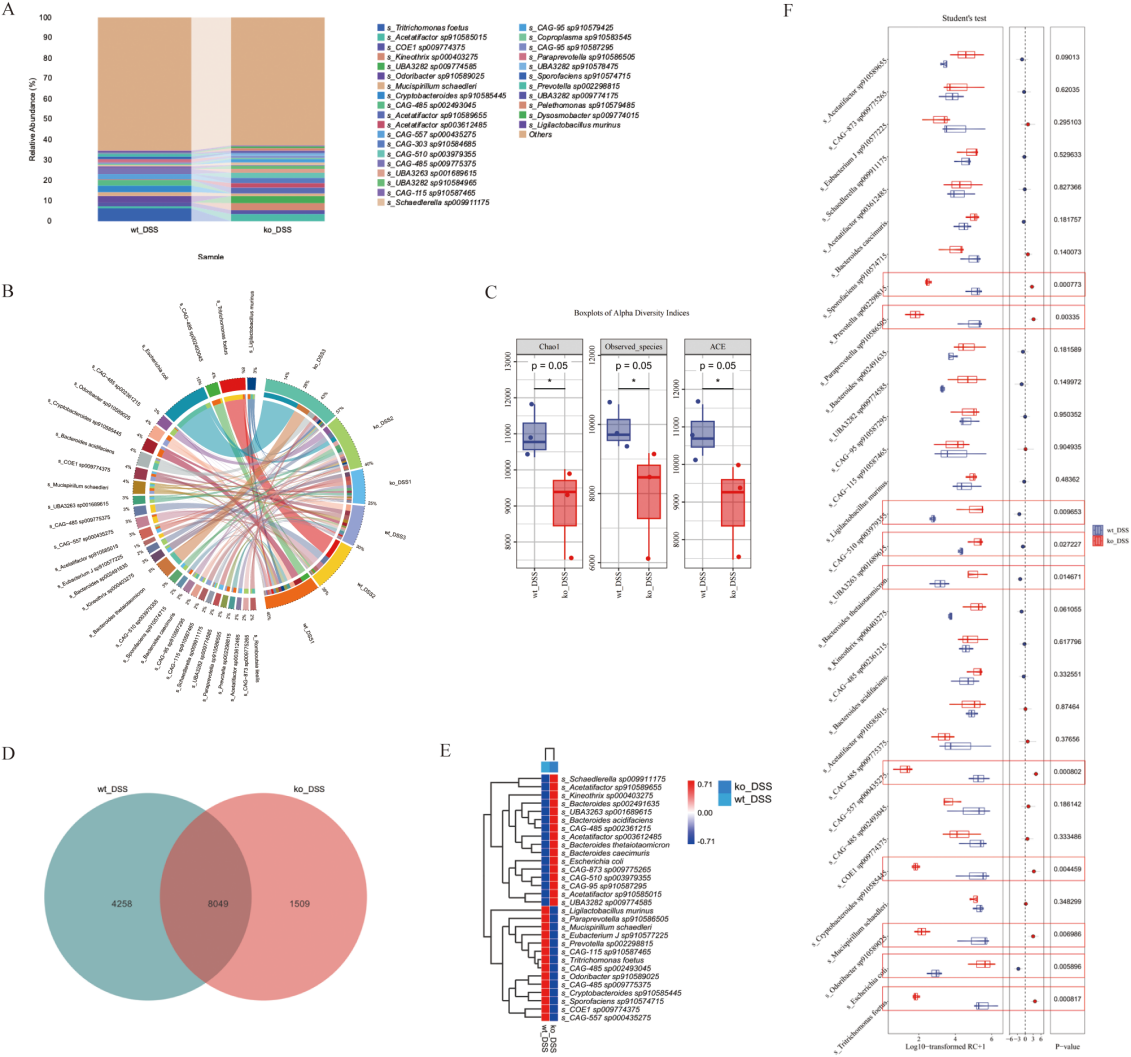
**(A)** Gut microbiota composition. The stacked bar chart shows significant variations in gut microbiota composition across 4 groups. **(B)** Beta diversity PCoA plot, the principal coordinate analysis (PCoA) plot reveals distinct clustering of gut microbiota communities among groups. **(C)** Alpha diversity. The boxplots display alpha diversity indices (Chao1, Good’s coverage, Simpson, and Pielou_e) across groups. **(D)** Differential species analysis. Heatmap, the heatmap on the right illustrates differentially abundant species between groups. Species showing significant differences are color-coded, with red and blue indicating higher and lower abundance, respectively. **(E)** Species differential analysis. The boxplots display a comparative analysis of gut microbiota differences among groups using the Kruskal-Wallis test. **(F)** Detailed taxonomic analysis. Stacked bar chart provides a more detailed view of gut microbiota composition at different taxonomic levels, revealing specific bacterial genera or families that are significantly altered in each group. *p < 0.05.

We performed metagenomic sequencing on fecal samples from four groups of mice: wt, wt_DSS, ko, and ko_DSS. Our analysis revealed distinct gut microbiota compositions among the groups. We focused on microbes with an abundance of 30. In the colitis model groups (wt_DSS and ko_DSS), the gut microbiota shifted notably. Specifically, Escherichia coli and Bacteroides increased significantly in the ko_DSS group, while *Tritrichomonas* was more abundant in the wt_DSS group. In the control groups, the gut microbiota also differed between the wt and ko groups. The ko group had higher abundances of *Limosilactobacillus* and *CAG-485* sp*002361215*, whereas the wt group showed higher levels of *Dubosiella*, *Akkermansia*, and *CAG-485* sp*002491945* (**Figure 2A**). A PCoA plot (**Figure 2B**) and a clustering heatmap (**Figure 2C**) both highlighted the distinct compositional differences between groups. Alpha diversity analysis (**Figure 2D**) indicated no significant differences in some indices, but notable changes in others, such as the trend in Chao1 diversity. Species differential analysis revealed that *Escherichia* levels varied significantly across groups (p < 0.05). While *Odoribacter* sp*910589025*, *Limosilactobacillus reuteri*, *Akkermansia muciniphila A*, *Ligilactobacillus murinus*, and *Tritrichomonas foetus* did not show statistical differences, their abundances changed markedly (**Figure 2E**). Finally, LEfSe analysis identified differentially abundant gut microbial taxa at various taxonomic levels, pinpointing specific bacterial genera or families that changed significantly in each group (**Figure 2F**).

This study primarily examines the alterations in gut microbiota composition between the wt_DSS and ko_DSS groups.

### 3.3 PD-L1 modulates gut microbiota composition in DSS-induced colitis mice

**Figure 3:**
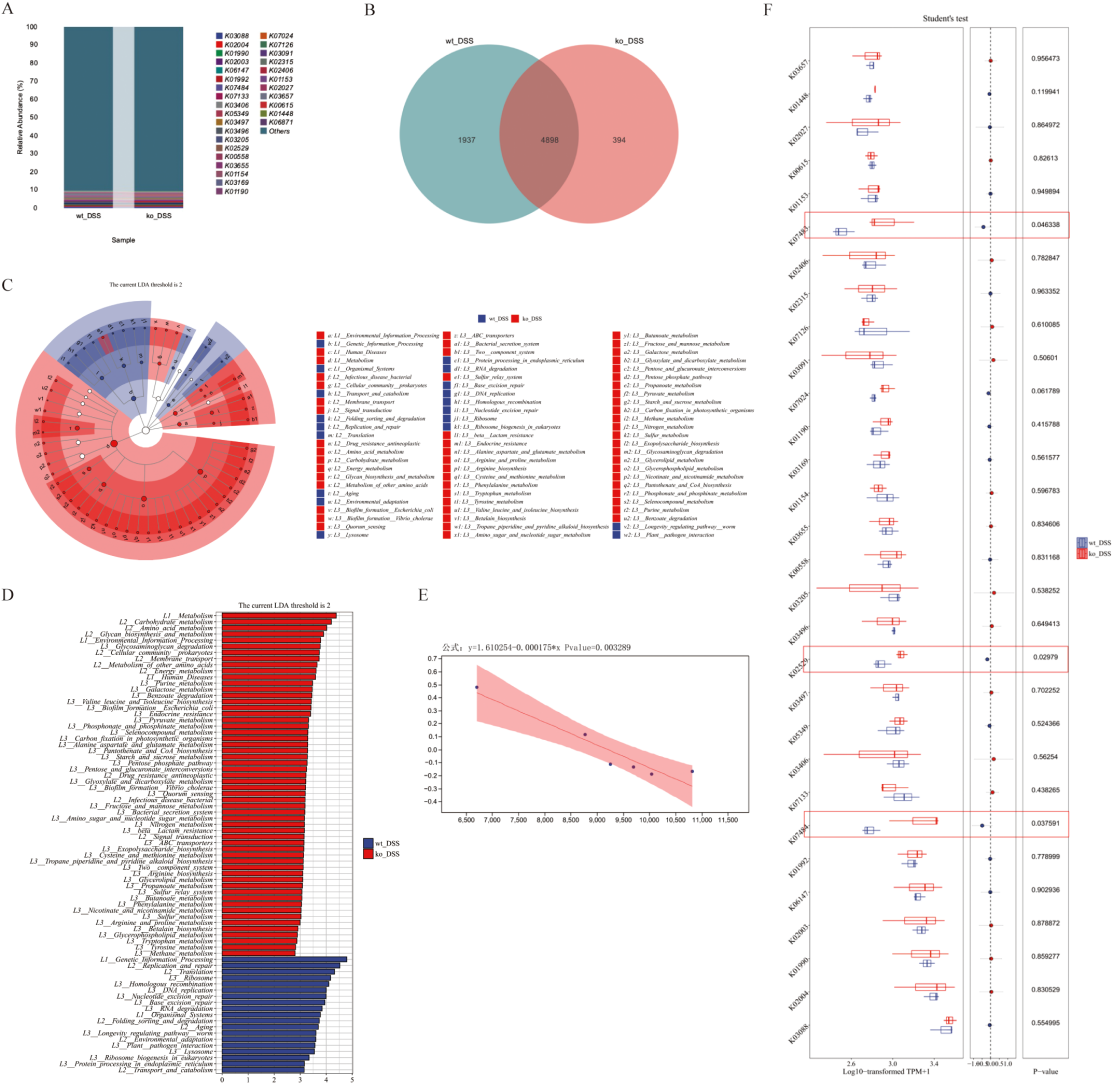
**(A)** Gut microbiota composition. The stacked bar chart shows the relative abundance of gut microbiota compositions between ko_DSS and wt_DSS groups. X-axis: Group names; Y-axis: Relative abundance of KEGG species levels. **(B)** Species differential analysis. This Circos Plot illustrates differentially abundant species between the two groups. Left: Abundance values (top 30); Right: Sample names. Colors in the inner ring mark species, and the outer ring shows their abundance. **(C)** Alpha diversity analysis. Boxplots indices (Chao1, Observed_species, ACE) across groups. Each panel corresponds to an alpha diversity index, as indicated in the grey area at the top. In each panel, the x-axis represents the group labels, and the y-axis represents the value of the corresponding alpha diversity index. In the boxplot, the elements are defined as follows: the top and bottom lines of the box represent the upper and lower quartiles (Interquartile Range, IQR); the central line denotes the median; the upper and lower whiskers indicate the maximum and minimum values (extremes within 1.5 times the IQR range); and points outside the whiskers represent outliers. The Kruskal-Wallis test was used to assess diversity indices, followed by Dunn’s test for *post hoc* analysis. A p-value less than 0.05 indicates statistically significant differences. **(D)** The composition Venn. The Venn diagram shows shared and unique species between ko_DSS and wt_DSS groups. Each colored block represents a group. Overlapping areas indicate shared species between groups, with numbers denoting species counts per region. **(E)** The composition heatmap. The groups are first clustered based on the similarity of their species abundance distribution, and then arranged horizontally according to the clustering results. Similarly, taxonomic units are clustered based on their distribution similarity across different groups and arranged vertically based on the clustering outcome. Red (0.71): High relative abundance in a group. White (0.00): Neutral/no significant abundance. Blue (-0.71): Low relative abundance or depletion. **(F)** The Boxplots Diagram. The diagram shows the species-level differential abundance analysis (normalized). Student’s t-test was applied for pairwise comparisons, with significant p-values marked. Red circles denote genes with statistically significant functional differences. DSS, Dextran sulphate sodium; ko, knockout; wt, wild-type.

Evaluation of gut microbiota in the DSS-induced colitis model revealed alterations in both the wt_DSS and ko_DSS groups (**Figures 3A, B**). The two groups shared 8,049 bacterial genera, while the wt_DSS and ko_DSS groups exhibited 1,509 and 4,258 unique genera, respectively (**Figure 3D**). Alpha diversity indices were used to assess the gut microbiota, showing significant differences in richness (Chao1, Observed species, and ACE) between the two groups (p = 0.05). However, no significant differences were observed in diversity (Simpson index), evenness (Pielou’s evenness), or coverage (Good’s coverage) (**Figure 3C**). The wt_DSS group showed higher enrichment of *Schaedlerella* sp*009911175*, *Acetatifactor* sp*910589655*, *Kineothrix* sp*000403275*, *Bacteroides* sp*00249163*, *UBA3263* sp*001689615*, *Bacteroides acidifaciens*, *CAG-485sp002361215*, *Acetatifactor* sp*003612485*, *Bacteroides thetaiotaomicron*, *Bacteroides caecimuris*, *Escherichia coli*, *CAG-873* sp*009775265*, *CAG-510* sp*003979355*, *CAG-95* sp*910587295*, *Acetatifactor* sp*910585015*, and *UBA3282* sp*009774585*. In contrast, *Ligilactobacillus murinus*, *Paraprevotella* sp*910586505*, *Mucispirillum schaedleri*, *Eubacterium J* sp*910577225*, *Prevotella* sp*002298815*, *CAG-115* sp*910587465*, *Tritrichomonas foetus*, *CAG-485* sp*002493045*, *Odoribacter* sp*910589025*, *CAG-485* sp*009775375*, *Cryptobacteroides* sp*910585445*, *Sporofaciens* sp*910574715*, *COE1* sp*009774375*, and *CAG-557* sp*000435275* were less enriched. The ko_DSS group exhibited the opposite trend (**Figure 3E**). Further differential species analysis identified significantly higher enrichment of *Escherichia coli* (p = 0.0058), *CAG-510* sp*003979355* (p = 0.010), *UBA3263* sp*001689615* (p 0.027), and *Bacteroides thetaiotaomicron* (p = 0.015). Conversely, *Tritrichomonas foetus* (p < 0.001), *CAG-557* sp*000435275* (p < 0.001), *Paraprevotella* sp*910586505* (p = 0.003), *Cryptobacteroides* sp*910585445* (p = 0.0044), *Odoribacter* sp*910589025* (p = 0.0069), and *Prevotella* sp*002298815* (p = 0.008) were significantly less enriched (**Figure 3F**).

### 3.4 PD-L1 alters gut microbiota functional units in DSS-induced colitis mice

**Figure 4:**
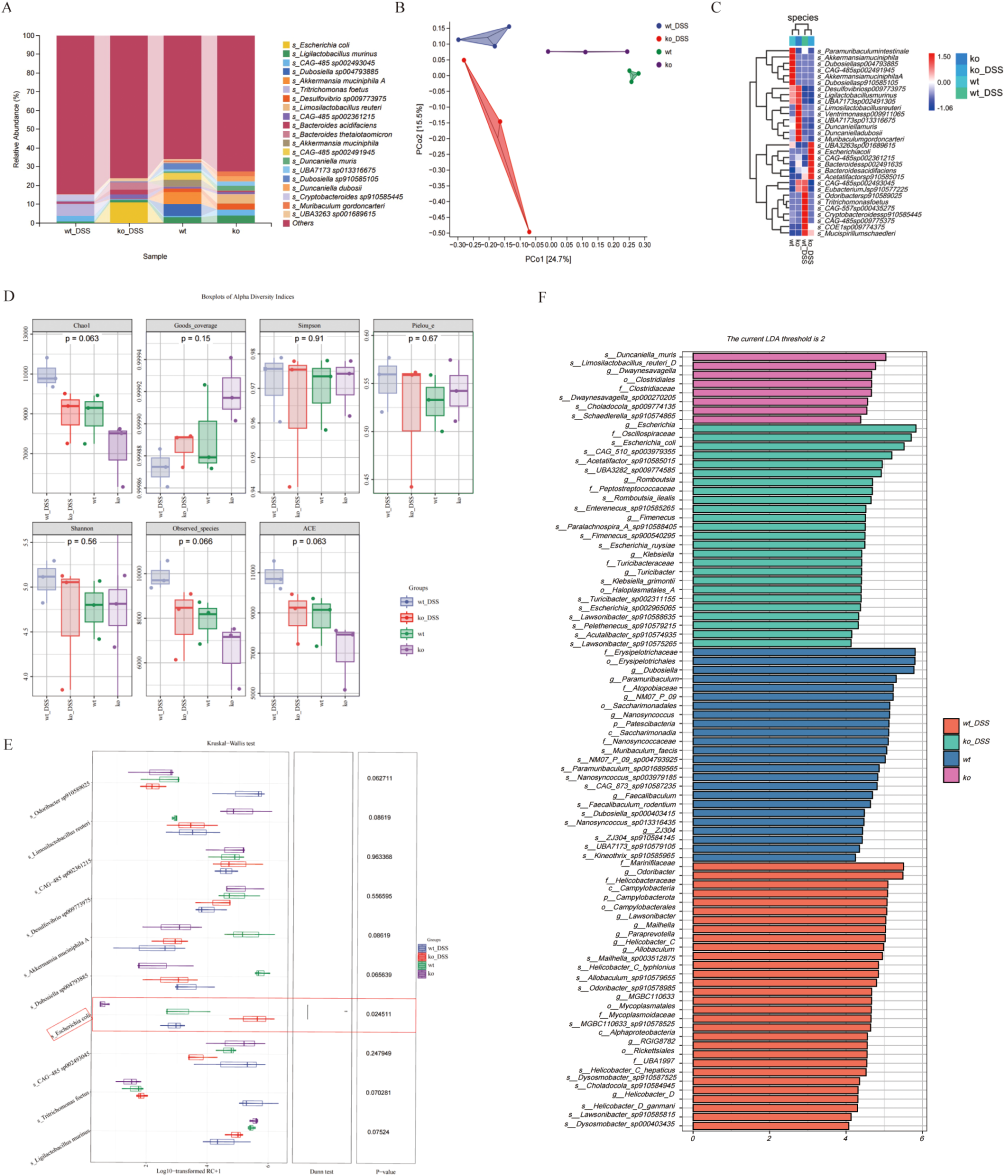
**(A)** Gut microbiota functional composition. The stacked bar chart shows the relative abundance of gut microbiota functional compositions between ko_DSS and wt_DSS groups. X-axis: Group names; Y-axis: Relative abundance of KEGG functional categories. Rows: KEGG Orthology (KO) identifiers representing microbial metabolic/enzymatic functions. Columns: groups (wt_DSS and ko_DSS). Values: Relative abundance (%) of each functional gene category. “Others”: Aggregated low-abundance functions. **(B)** The functional Venn. The Venn diagram shows shared and unique species between ko_DSS and wt_DSS groups. Each colored block represents a group. Overlapping areas indicate shared functional units between groups, with numbers denoting functional unit counts per region. **(C)** The Circular branching Diagram. Cladogram of LEfSe-identified functional pathways in ko_DSS (red) and wt_DSS (green) mice. Node size reflects LDA effect size (threshold > 2). Key pathways are annotated (see Results). **(D)**The bar plot. This bar plot displays the functional units with statistically significant differences between the ko_DSS and wt_DSS groups, as determined by Linear Discriminant Analysis (LDA) effect size (LEfSe). Y-axis: Functional units showing significant intergroup differences (p < 0.05). X-axis: LDA score (log10-transformed), representing the magnitude of differential abundance. Longer bars indicate stronger discriminatory power. Red: Functional units enriched in ko_DSS. Green: Functional units enriched in wt_DSS. **(E)** Linear Regression Plot. This plot shows the relationship between two variables, with a fitted regression line and a shaded confidence interval. The equation and p-value indicate the strength and significance of the relationship. **(F)** The Boxplots Diagram. The diagram shows the statistical comparison of functional gene expression (KEGG Orthology, KO terms) between ko_DSS and wt_DSS groups. KO Terms: Listed rows represent microbial metabolic/pathway genes. Expression Values: Log10-transformed TPM+1 (Transcripts Per Million, normalized and log-transformed).Student’s t-test p-values for each KO term. Group name: wt_DSS vs. ko_DSS. Red circles denote genes with statistically significant functional differences. DSS, Dextran sulfate sodium; ko, knockout; wt, wild-type.

We next performed functional analysis of the gut microbiota in colitis model groups. The intergroup differences in microbial composition significantly altered gene functional profiles. Statistical analysis of feature tables using the KEGG database enabled visualization of compositional distribution at functional classification levels (top 30 categories), presented as a bar chart (**Figure 4A**). Comparative analysis revealed 4,898 shared functional genes between groups, with 394 and 1,937 genes uniquely identified in the ko_DSS and wt_DSS groups, respectively (**Figure 4B**). Subsequently, LEfSe (LDA Effect Size) analysis was employed to rank differentially enriched functional pathways between ko_DSS and wt_DSS groups. Results demonstrated that the ko_DSS group exhibited significant enrichment of pathways associated with pro-inflammatory and pathogenic functions, including: Bacterial secretion systems (Type III/IV), linked to virulence traits in *Escherichia coli* and *CAG taxa.* ABC transporters (e.g., K02003/K02004) and drug resistance pathways, suggesting enhanced antibiotic resistance in ko_DSS. Immune dysregulation-related pathways, such as the lysosome pathway (associated with heightened phagocytic activity due to inflammation) and toxification/detoxification (reflecting oxidative stress in chronic colitis). In contrast, the wt_DSS group showed enrichment of pathways supporting anti-inflammatory and metabolic homeostasis, including: Short-chain fatty acid (SCFA) metabolism (e.g., butyrate synthesis via K01990), which promotes Treg differentiation and epithelial barrier integrity. Carbohydrate metabolism (e.g., glycosyl hydrolases), driven by dietary fiber fermentation from *Ligilactobacillus* and *Bacteroides*. Ribosome biosynthesis, indicative of stable microbiome-protein synthesis. Commensal-mucosal crosstalk pathways (e.g., arginine/proline metabolism) modulating nitric oxide (NO) production and immune signaling (**Figures 4C, D**). Further normalization of functional unit abundances revealed statistically significant enrichment of pro-inflammatory/virulence-associated genes in ko_DSS (e.g., K07133 [Type III secretion system, p = 0.038], K02529 [uncharacterized protein, p = 0.030], and K01190 [glycoside hydrolase, p = 0.046]). The wt_DSS group harbored higher abundances of anti-inflammatory/commensal genes (e.g., K01990 [acetyl-CoA synthetase] and K01153 [glycosyl hydrolase]), though these lacked statistical significance (**Figure 4F**). Finally, linear regression analysis of functional and taxonomic correlations identified a significant negative association (p = 0.003) between an immune variable (x; e.g., inflammatory cytokine levels) and microbial/metabolic responses (y; e.g., beneficial taxon abundance), underscoring PD-L1’s role in microbiota-immune crosstalk during colitis (**Figure 4E**).

### 3.5 PD-L1 regulates gut microbiota genes profiles in DSS-induced colitis mice

**Figure 5:**
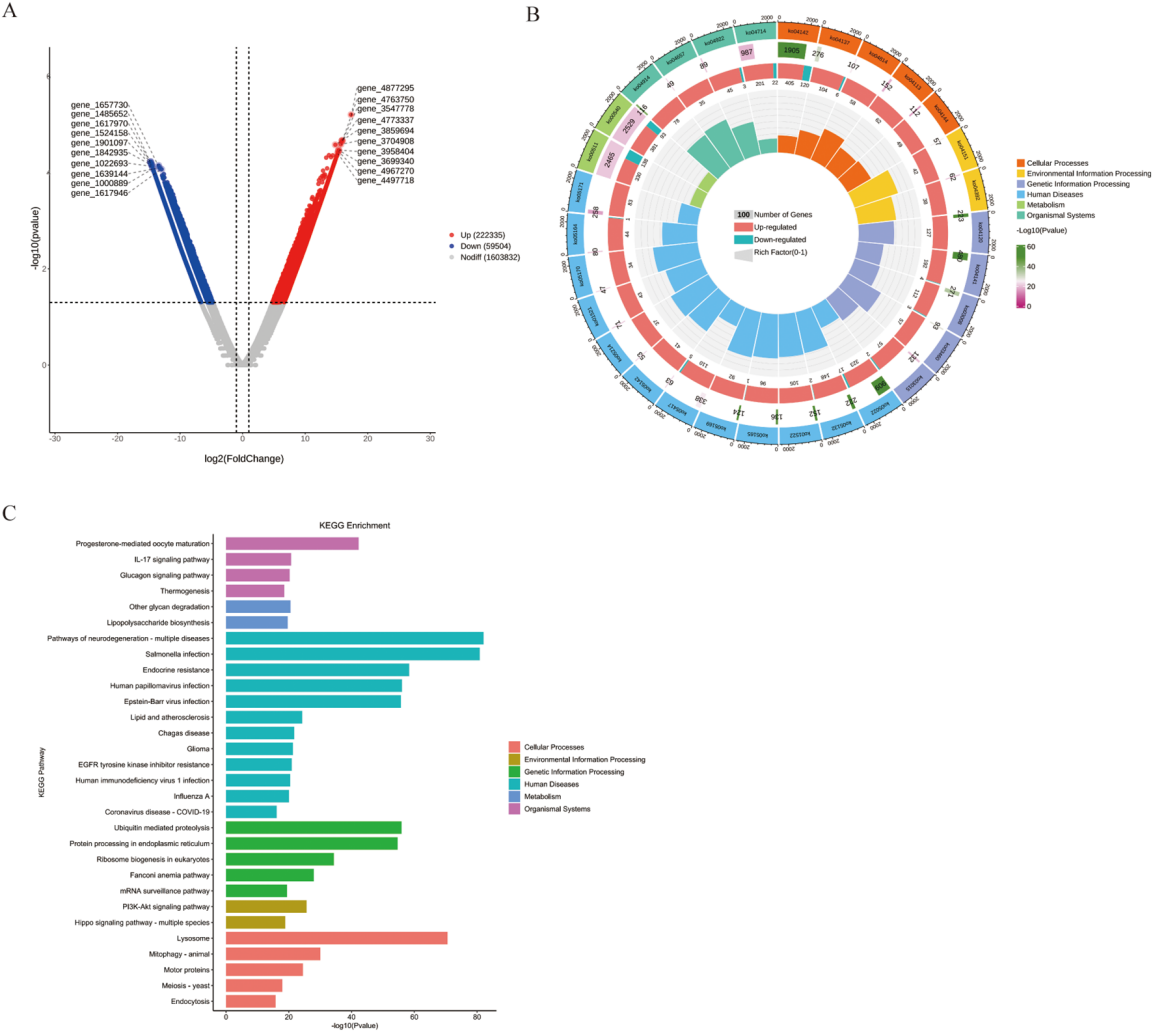
**(A)** The volcano plot. This volcano plot displays the differentially abundant genes between the ko_DSS and wt_DSS groups. The x-axis represents the log2 fold change, and the y-axis represents the negative logarithm of the adjusted p-value (-log10(Adjust)). Genes with a fold change greater than 2 and an adjusted p-value less than 0.05 are considered significantly differentially abundant. Red dots represent upregulated genes in the ko_DSS group, blue dots represent downregulated genes, and grey dots represent genes without significant changes. The plot highlights specific genes that are differentially expressed between the two groups. **(B)** The Circular Tree Diagram. This circular tree diagram presents a hierarchical clustering of gut microbiota functions across different categories: Outer ring: major KEGG categories with bar length reflecting pathway significance (-log_10_(p-value)). Inner tracks: gene counts (grey), regulation direction (red/blue), and Rich Factor (yellow). Highlighted immune pathways (e.g., Toll-like receptor signaling) suggest altered inflammatory responses in ko_DSS. **(C)** The KEGG Enrichment Analysis. This bar plot illustrates the enrichment of various KEGG pathways in the ko_DSS and wt_DSS groups. Each bar represents a specific pathway, with the x-axis showing the negative logarithm of the adjusted p-value (-log10(Adjust)), indicating the significance of enrichment.

Finally, we performed gene-level differential analysis of fecal samples from the mouse model. Compared to the wt_DSS group, ko_DSS group exhibited 222,335 genes upregulated and 59,504 genes downregulated genes (**Figure 5A**). KEGG enrichment analysis of these differentially expressed genes (DEGs) revealed significant associations with immune/inflammatory pathways in ko_DSS, including:IL-17 signaling, Lipopolysaccharide biosynthesis, Endocrine resistance. Additionally, infection/disease-related pathways were enriched, such as COVID-19, HIV infection and Salmonella infection. Notably, metabolic and cellular dysregulation pathways, including thermogenesis and lysosome, were also altered (**Figures 5B, C**), suggesting shifts in response to DSS-induced inflammation in *Pdcd1lg1* deficient mice.

## 4 Discussion

The study provides compelling evidence that PD-L1 deficiency exacerbates DSS-induced colitis through profound alterations in gut microbiota composition and function. PD-L1 knockout mice exhibited more severe clinical symptoms, such as marked weight loss, colon shortening, and elevated DAI scores, which were accompanied by distinct changes in the microbial community. These changes were characterized by an increase in pathogenic taxa, including *Escherichia coli* and *CAG-510*, and a decrease in beneficial species, such as *Tritrichomonas foetus* and *Ligilactobacillus murinus*. Notably, metagenomic analysis revealed PD-L1-dependent shifts in microbial virulence pathways and metabolic functions. These findings align with emerging understanding of PD-1/PD-L1 axis in mucosal immunity, while revealing novel microbiota-mediated mechanisms.

The PD-1/PD-L1 pathway is regarded as a potential therapeutic target in IBD. Studies reveal that PD-1 and PD-L1 expression is significantly upregulated in inflamed intestinal mucosa, primarily localized to non-ulcerated regions. This spatial heterogeneity suggests that PD-1/PD-L1 may protect the mucosal barrier by suppressing localized hyperimmune responses, while their loss in ulcerated zones could contribute to uncontrolled inflammation (34). Meanwhile, PD-L1 maintains intestinal homeostasis by modulating dendritic cells (e.g., XCR1+ DCs) and Tregs (35). Since this study primarily focuses on analyzing the impact of gut microbiota changes on colitis, we observed that the PD-L1 knockout mice showed marked depletion of mucin-protective species (*Akkermansia muciniphila*) and SCFA-producers (*L. murinus*), while harboring increased mucolytic bacteria (*Bacteroides thetaiotaomicron*). The observed imbalances likely impair epithelial integrity, as demonstrated by histopathological evidence of severe edema and ulceration, accompanied by downregulation of tight junction genes (including occludin and claudins) and upregulation of bacterial invasion pathways such as the Type III secretion system. Together, these pathological changes collectively disrupt intestinal barrier function.

The functional analysis revealed significant metabolic reprogramming characteristics. Butyrate (a SCFA) maintains intestinal homeostasis by serving as an HDAC inhibitor, promoting Treg differentiation via epigenetic modulation of the Foxp3 locus while suppressing Th17 responses through IL-6/STAT3 inhibition (36–38). Downregulation of butyrate kinase (K01990) aligns with reduced butyrate-producing bacteria (e.g., *Faecalibacterium prausnitzii*) in IBD, exacerbating inflammation in this research. Gram-negative bacterial lipopolysaccharide (LPS) activates the TLR4-NF-κB-IL-23/Th17 signaling axis while simultaneously directly compromising intestinal tight junction integrity (39, 40). This pathophysiological mechanism is highly consistent with our experimental findings demonstrating significant downregulation of occludin and claudin expression in the intestinal epithelium. These results align with clinical observations of elevated plasma LPS concentrations in IBD cases (41).

Research evidence demonstrates that the gut microbiota can significantly modulate the therapeutic efficacy of PD-1/PD-L1 inhibitors in cancer treatment (42). Specifically, in melanoma and colorectal cancer patients who are non-responders to anti-PD-1 therapy, studies have observed abnormal proliferation of Bacteroides species and significant depletion of beneficial bacteria such as *Akkermansia muciniphila* (43, 44). For the first time, our study has replicated these key findings in a PD-L1-deficient mouse model: not only confirming reduced abundance of *Akkermansia muciniphila*, but more importantly, functional predictive analysis revealed significant upregulation of antibiotic resistance-related genes including ABC transporters (K02003/K02004), β-lactamase resistance genes, and multidrug efflux systems. This groundbreaking discovery suggests that deficiency in PD-L1 signaling may promote antibiotic resistance by altering the functional profile of gut microbiota—a finding with critical implications for current antibiotic-based clinical treatment strategies for IBD.

In PD-L1 knockout mice, besides an increase in pathogenic *Escherichia coli*, there is also a rise in *CAG-510* and *UBA3263* sp*001689615*. However, the functions and mechanisms of these two bacterial species have not yet been characterized. They may serve as biomarkers in the PD-L1 knockout mice colitis model and could be involved in the PD-L1 signaling pathway, exacerbating intestinal inflammation.

Certainly, our study has limitations regarding the relatively small sample size. However, based on the current analytical framework, we aim to further investigate the therapeutic potential of fecal microbiota transplantation using these differentially altered microbial communities for IBD treatment in subsequent research.

## Data Availability

The datasets presented in this study can be found in online repositories. The names of the repository/repositories and accession number(s) can be found below:https://www.ncbi.nlm.nih.gov/, PRJNA1258551https://www.ncbi.nlm.nih.gov/, PRJNA1261602.
